# A quantitative transcriptomic analysis of the physiological significance of mTOR signaling in goat fetal fibroblasts

**DOI:** 10.1186/s12864-016-3151-y

**Published:** 2016-11-07

**Authors:** Yuting Fu, Xu Zheng, Xiaoyang Jia, Uyanga Binderiya, Yanfeng Wang, Wenlei Bao, Lili Bao, Keyu Zhao, Yu Fu, Huifang Hao, Zhigang Wang

**Affiliations:** 1 College of Life Sciences, Inner Mongolia University, Hohhot, 010021 China; 2 Clinical Laboratory, The Hulunbuir People’s Hospital, Hailaer, 021008 China; 3 College of Basic Medical Science, Inner Mongolia Medical University, Hohhot, 010021 China

**Keywords:** Goat, RNA-Seq, Differential expression genes, mTOR

## Abstract

**Background:**

Mammalian target of rapamycin (mTOR) is an evolutionarily conserved serine/threonine kinase that is a central regulator of cell growth and metabolism. CCI-779 is a specific inhibitor of the mTORC1 signaling pathway.

**Results:**

We performed comparative transcriptome profiling on Inner Mongolia Cashmere goat fetal fibroblasts (GFbs) that were treated with CCI-779 and untreated cells. A total of 365 differentially expressed genes (DEGs) appeared between untreated and CCI-779-treated GFbs, with an FDR ≤0.001 and fold-change ≥2. These 365 DEGs were associated with mTOR signaling; 144 were upregulated in CCI-779-treated cells, and 221 were downregulated.

Additionally, 300 genes were annotated with 43 Gene Ontology (GO) terms, and 293 genes were annotated with 194 Kyoto Encyclopedia of Genes and Genomes (KEGG) pathways. Three RNA polymerase II and polymerase III subunits, 3 transcription factors, and 5 kinases in mTOR signaling were differentially expressed in CCI-779-treated GFbs. Further 6 DEGs were related to amino acid metabolism, 11 mediated lipid metabolism, 11 participated in carbohydrate metabolism, and 5 were involved in single-nucleotide metabolism. Based on our quantitative transcriptomic analysis, 40 significant DEGs with important function related to metabolism, RNA polymerase, transcription factors and mTOR signaling were selected for qPCR analysis, and the quantitative results between the two analysis methods were concordant. The qPCR data confirmed the differential expression in the RNA-Seq experiments. To validate the translational significance of the findings in certain differentially expressed genes, S6K1 and VEGF were detected by western blot, and these two proteins showed a differential expression between non-treated and treated with CCI-779 groups, which were consistent with mRNA abundance. The data showed a preliminary significance of the findings in the protein levels.

**Conclusions:**

CCI-779 induces transcriptomic changes, and mTOR signaling might have significant function in the activation of RNA polymerase and certain transcription factors and in the metabolism of amino acids, lipids, carbohydrates, and single nucleotides in GFbs. These data filled the vacancy in the systematical profiling of mTOR signaling on Cashmere goat fetal fibroblasts.

**Electronic supplementary material:**

The online version of this article (doi:10.1186/s12864-016-3151-y) contains supplementary material, which is available to authorized users.

## Background

Mammalian target of rapamycin (mTOR) is an evolutionarily conserved serine/threonine kinase that belongs to the phosphatidylinositol kinase-related kinase (PIKK) family. mTOR was renamed mechanistic target of rapamycin (MTOR) by the Human Genome Organization (HUGO) Gene Nomenclature Committee (HGNC). mTOR exists as 2 physically and functionally distinct protein complexes, mTORC1 and mTORC2; is a central regulator of cell growth and metabolism; and is sufficient to induce specific metabolic processes [1]. Pharmacological and genetic studies have demonstrated that mTORC1 activation influences cell growth and metabolism in various organisms, from yeast to human [2]. mTORC1 inhibition decreases protein synthesis and gene transcription. The mTOR signaling pathway is emerging as a significant regulator of gene transcription, governing various cellular processes [3]. It is likely that the physiological significance of mTOR signaling will expand. mTORC1 has a distinct function in cell metabolism, depending on the environmental nutrient status [4]. With sufficient nutrients, mTORC1 positively regulates anabolic processes, including protein synthesis, lipid synthesis, ribosome biogenesis, and nucleotide biosynthesis [5]. De novo nucleotide biosynthesis is an approach for providing building blocks for DNA and RNA. Increasing evidence from functional genomics studies suggests that mTORC1 signaling promotes nucleotide biosynthesis [6]. Recent studies have demonstrated that mTORC1 signaling post-translationally regulates de novo pyrimidine synthesis through its downstream target ribosomal protein S6 kinase 1 (S6K1), which phosphorylates S1859 of CAD (carbamoyl-phosphate synthetase 2, aspartate transcarbamoylase, dihydroorotase), the enzyme that catalyzes the 3 three steps of de novo pyrimidine synthesis [7, 8]. New pyrimidine synthesis can accommodate increased DNA and RNA synthesis.

Lipid biosynthesis is essential for maintaining cellular homeostasis. mTOR signaling has a fundamental function in regulating various aspects of lipid metabolism in mammalian cells, including lipogenesis, adipogenesis, lipolysis, and lipid oxidation [9, 10]. mTORC1 promotes de novo lipogenesis through the activation of SREBP-1 (sterol regulatory element-binding protein) and PPAR-γ (peroxisome proliferator-activated receptor) [11, 12]. Moreover, inhibition of mTOR signaling promotes lipolysis, thermogenesis, and lipid storage [13–15]. mTOR signaling is important in the maintenance of systemic lipid homeostasis.

It is believed that mTORC1 has a significant function in energy homeostasis in a cell-autonomous [16]. In many cell systems, mTORC1 couples PI3K (phosphoinositide 3-kinase) and PKB (protein kinase B) to control glucose uptake [17, 18] and glycosis [19, 20]. mTORC1 is linked to the control of glycolysis through 2 transcription factors, c-Myc and HIF (hypoxia-inducible factor) 1α, each of which upregulates glycolytic enzymes and glucose transporters [21]. Further, mitochondria are the main site of ATP production, and mTOR signaling is associated with the activation of mitochondria and ATP synthase [22, 23].

mTORC1 activity depends strictly on sufficient levels of intracellular amino acids, because amino acids are essential for mTORC1 activity [24], leucine is believed to promote energy metabolism, including glucose uptake, mitochondrial biosynthesis, and fatty acid oxidation, to provide energy for protein synthesis while inhibiting protein degradation [25]; yet, the function of mTOR in amino acid catabolism remains unknown.

mTORC1 governs transcription, as first characterized in yeast, and mTORC1 inhibition also affects the expression of important genes in mammalian cells [26]. mTORC1 alters gene expression by impacting the activity of transcription factors, including STAT3, TFEB, NRF1, HIF1α, and YY1-PGC1α [27]. Further, mTOR signaling governs the synthesis of Pol I-transcribed 45S rDNA and Pol III-transcribed genes, including tRNA and 5S rRNA, by binding to the promoters of RNA polymerase I- and III-transcribed genes [28]. mTOR associates with TFIIIC, a DNA-binding factor that resides in tRNA and 5S rRNA genes, and targets their repressor, Maf1 [29]. The mTOR kinase is required for the regulation of Maf1 phosphorylation and the control of RNA polymerase III-dependent transcription [30]. mTOR might be involved in the activities of all 3 RNA polymerases.

RNA-Seq generates deep sequencing data for the direct quantification of transcripts. Integrative transcriptome analysis was performed to decipher the molecular mechanism underlying rapamycin-induced anti-tumor and anti-angiogenic effects, and a transcriptional network was discovered to be enriched for genes that were related to angiogenesis and extracellular matrix remodeling [31]. RNA-Seq revealed the activation of an aurora kinase-driven mTOR pathway in patients with sarcomatoid metastatic renal cell carcinoma [32]. These studies are helpful in examining the regulation of cancer behavior that is mediated by mTOR signaling. By high-throughput RNA-Seq, many genes that were related to apoptosis and migration, which enhance CTL survival into memory, were identified in mouse CTLs in which mTOR was inhibited [33]. Profiling of the fetal and adult rat liver transcriptome confirmed that mTOR signaling in liver has distinct physiological functions in the adult and fetus [34]. Using RNA-Seq to find regulatory networks and novel regulators can help us to decipher mTOR signaling.

The mTORC1 signaling pathway has been extensively examined in human, mouse, and rat but not in cashmere goat, due to the lack of basic data in this animal. To study the physiological significance of mTORC1 signaling in Inner Mongolia Cashmere goat cells, we used RNA-Seq to examine genome-wide gene expression differences in cashmere goat fetal fibroblasts in which mTORC1 activation was inhibited. In our previous studies, treatment of goat fetal fibroblasts with CCI-779 inhibited mTOR signaling and proliferation in vitro [35]. CCI-779, also called temsirolimus or rapamycin 42-[2,2-bis (hydroxymethyl) propionate], is an ester of rapamycin and a specific inhibitor of mTORC1. In the current study, we used CCI-779 to treat cashmere goat fetal fibroblasts and to examine genome-wide gene expression differences. Differentially expressed genes (DEGs) compared to untreated cells were identified from a whole-transcriptome background. Our data suggest that the expression of RNA polymerase subunits; certain transcription factors; and important enzymes in amino acid, lipid, carbohydrate, and single nucleotide metabolism is regulated by mTORC1 in goat fetal fibroblasts.

## Methods

### Cell culture conditions and design of experiment

**Fig. 1:**
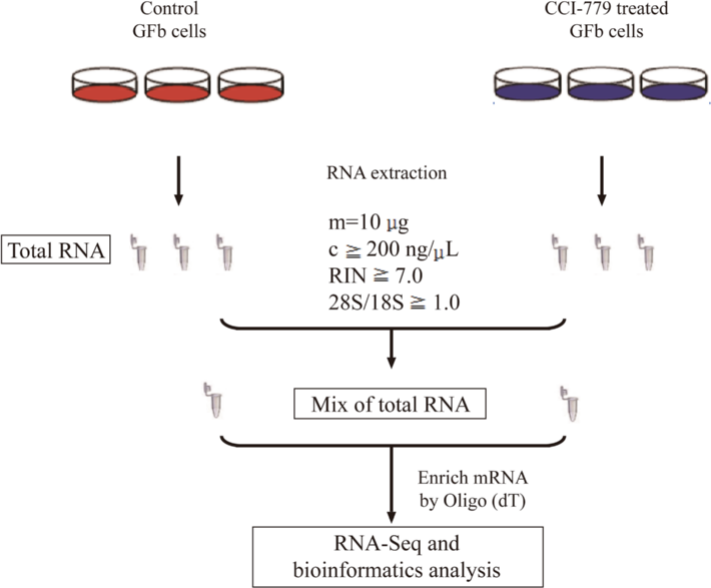
Overall design of experiment. Inner Mongolia Cashmere goat fetal fibroblasts (GFbs) were maintained as monolayer cultures in Dulbecco modified Eagle’s medium (DMEM)/F12, supplemented with 10 % fetal bovine serum and penicillin/streptomycin. Untreated GFbs and GFbs that were treated with 50 nM CCI-779 (temsirolimus) for 12 h were cultured in petri dishes (diameter = 100 mm), and 5.8 × 10^6^ cells were collected for each sample. The cells processing were repeated 3 times and to isolate total RNA separately. Then, the 3 isolated total RNAs were mixed for RNA-seq

Inner Mongolia Cashmere goat fetal fibroblasts (GFbs) were maintained as monolayer cultures in Dulbecco modified Eagle’s medium (DMEM)/F12 (Gibco, Paisley, PA49RF, Scotland, UK), supplemented with 10 % fetal bovine serum (FBS, Hyclone Laboratories, Inc. Logan, UT, USA) and penicillin/streptomycin (Sigma-Aldrich, Inc. St. Louis, USA). Cell cultures were maintained and incubated at 37 °C in humidified air with 5 % CO_2_. The P2 ~ P4 cells were used for all experiments. Untreated GFbs and GFbs treated with 50 nM CCI-779 (temsirolimus) for 12 h were cultured in petri dishes (diameter = 100 mm), respectively, and 5.8 × 10^6^ cells were collected for each sample. The cells processing were repeated 3 times and to isolate total RNA, separately. Then, the 3 isolated total RNAs were mixed for RNA-seq (Fig. 1). CCI-779, a derivative of rapamycin, was synthesized by Selleck Chemicals LLC (Huston, Texas, USA) and dissolved in DMSO (Sigma Chemical Corp., St. Louis, MO). The concentration of DMSO in the final solution was 0.5 % (v/v).

### RNA preparation

Total RNA from control and CCI-779-treated GFbs was extracted using RNAzol (RNAiso Plus, TaKaRa Co. Lts., China) per the manufacturer’s instructions. RNA samples were then digested with RNase-free DNase I to eliminate residual genomic DNA, and the digestion products were purified using magnetic beads. The total RNA that had a standard of concentration ≥ 200 ng/μl, mass ≥ 10 μg, 28 S/18 S ≥ 1.0, and RNA integrity number (RIN) ≥ 7.0, as measured on an Agilent 2100 Bioanalyzer (Agilent technologies Inc., Santa Clara, CA, USA), was subjected to RNA-Seq.

### cDNA library construction and sequencing

mRNA for each sample was enriched using oligo (dT) magnetic beads, mixed with fragmentation buffer, and fragmented (approximately 200 bp). Then, first-strand cDNA was synthesized using random hexamer-primed reverse transcription, and second-strand cDNA was synthesized. The integrity and size were checked on an Agilent 2100 Bioanalyzer (Agilent technologies Inc., Santa Clara, CA, USA), and the cDNA library was sequenced on an Ion Proton platform (Life Technologies, Carlsbad, CA, USA).

### Sequence data processing and alignment with reference

The sequencing-obtained raw image data were transformed into sequence data, called raw data or raw reads, which were deposited in the SRA (http://www.ncbi.nlm.nih.gov/sra/) database under the submission number of SRP085885. Clean reads were obtained by trimming the adaptor sequences and removing ambiguous nucleotides, low-quality sequences, contaminated microbial sequences, and ribosomal RNA sequences. SOAP2 [36] was used to align all clean reads to the reference gene (http://ftp.ncbi.nih.gov/genomes/Capra_hircus/RNA/) and reference genome (http://ftp.ncbi.nih.gov/genomes/Capra_hircus/Assembled_chromosomes/seq/). Then, the length distribution, alignment statistics, sequencing saturation, randomness assessment, and distribution of reads against the reference gene and genome were analyzed.

### Screening and functional analysis of differentially expressed genes

The expression level of each gene was determined by the number of reads that was uniquely mapped to a specific gene and the total number of uniquely mapped reads in the samples. Expression level was calculated using the RPKM algorithm (reads per kb per million reads) [37]. The RPKM method eliminates the influence of gene length and sequencing discrepancies in calculating expression. Thus, RPKM values can be used directly to compare differences in expression between samples.

Differentially expressed genes (DEGs) were screened, based on Poisson distribution analysis [38], a strict algorithm that identifies DEGs between 2 samples. Genes were considered to be differentially expressed if they met the following criteria: fold-change ≥ 2 and FDR ≤ 0.001. The functional characteristics of DEGs were analyzed by pairwise comparison. Using BLAST (2.2.23) and Blast2GO (2.2.5) [39], the DEGs were provided with functional annotations, including Gene Ontology (GO) and Kyoto Encyclopedia of Genes and Genomes (KEGG). Cluster (3.0) [40] and Java TreeView (1.1.6r2) were used to perform cluster analysis of gene expression patterns. GO terms that met a corrected *p*-value ≤ 0.05 were defined as significantly enriched GO terms. The nr database, compiled by the NCBI (http://www.ncbi.nlm.nih.gov/) as a comprehensive protein database for BLAST searches, was used Blast2GO to obtain the GO annotation of DEGs. WEGO [41] was used to perform a functional classification of the DEGs and understand the distribution of gene functions on a macro level. Pathway enrichment analysis was conducted with the KEGG database [42].

### Quantitative real-time PCR

A total of 40 genes were selected for quantitative real-time PCR analysis, with the housekeeping gene *β-actin* as a reference. *β-actin* was amplified with the following primer pair: forward 5′-CCACTGGCATTGTCATGGACTC-3′ and reverse 5′- TTCCTTGATGTCACGGACGATTT-3′. Primers for all genes are listed in Additional file 1. Total RNA from two groups’ composites (one RNA composite from untreated cells and one from treated cells) was reverse-transcribed with an oligo (dT)_12–18_ primer using the AMV 1st Strand cDNA Synthesis Kit (Takara Co. Ltd., China). qPCR was performed on a Bio-Rad Chromo 4 PCR System using SYBR® Premix Ex Taq™ (Perfect Real Time) (TaKaRa Co. Ltd., China); 1 μL cDNA was amplified in a 25-μl reaction, containing 10 mol/L forward primer (0.5 μl), 10 mol/L reverse primer (0.5 μl), 2 × SYBR® Premix Ex Taq™ (12.5 μl), and nuclease-free water (10.5 μl) with the following program: 95 °C for 5 min; 40 cycles of 95 °C for 15 s, 54 °C for 30 s, and 72 °C for 30 s; and 72 °C for 10 min. To get reliable calculation of statistical significance, 3 samples per group and 3 technical replicas for per samples were performed for per differentially expressed gene. 2^-ΔΔCT^ values were calculated to determine expression levels. The qPCR results were analyzed by Student’s T test to compare expression between 2 groups.

### Western blot analysis

GFb cells were plated onto 55 cm^2^ petri dishes at 3 × 10^5^ per dish and incubated. Subconfluent cells (about 5.5 × 10^6^) were treated with 50 nM CCI-779 (temsirolimus) for 12 h, collected with trypsin, washed 3 times with cold PBS, and lysed in buffer that contained 20 mM Tris (pH 8.0), 137 mM NaCl, 100 g/L glycerol, 50 g/L Triton X-100, 2 g/L Na_2_VO_4_, and 4 g/L EDTA; 10 ml PMSF (0.1 M) and 10 ml ALT (10 g/L) were added per 1 ml lysis buffer immediately before use. The cell lysates were put on ice for 15 min and centrifuged at 15,000 rpm at 4 °C for 20 min, and the supernatant was transferred to new tubes.

The concentrations of the lysates were measured by Coomassie Plus (Bradford) Assay (Thermo Scientific). Equal amounts (40 μg) of protein were electrophoresed on 12 % (w/v) sodium dodecylsulfate polyacrylamide gels. Proteins were transferred to Hybond-polyvinylidene difluoride membranes (Amersham) and incubated with the primary antibodies overnight at 4 °C and peroxidase-conjugated secondary antibodies at room temperature for 1 h. Enhanced chemiluminescence (ECL) (Amersham) was used to detect the signals.

### Statistical analysis

Descriptive statistics were generated for all quantitative data, expressed as mean ± SD. The mean ± SD values were calculated from 3 samples per group and 3 technical replicas for per samples. Differences in means between control and CCI-779-treated groups were determined by Student’s T test. All statistical analysis were performed using GraphPad Prism, v.5.00 (GraphPad Software, CA, USA).

## Results

### RNA-Seq data processing, alignment, and assessment

RNA was isolated from control cells (Non-treated) and 50 nM CCI-779-treated cells for RNA sequencing analysis. By sequencing, 15,138,753 and 18,411,100 raw reads were generated for the control and CCI-779-treated groups, respectively. After the removal of adaptor sequences, ambiguous nucleotides, low-quality sequences, contaminated microbial sequences, and ribosomal RNA sequences, a total of 14,689,607 and 17,861,831 clean reads with lengths of 120–150 bp from 2 sequencing libraries were gathered for further analysis. Clean reads of 2 samples were aligned with TMAP {version 3.4.1} to the goat reference genes and genome, and uniquely aligning reads were summated to generate read count-based gene expression estimates. The mapping ratio to the reference genes ranged from 86.30 to 86.98 %, and above 80 %, whereas the mapping ratio to the reference genome ranged from 98.14 to 98.35 %, exceeding 97 %.

### Analysis of differentially expressed genes related to mTOR signaling

**Fig. 2:**
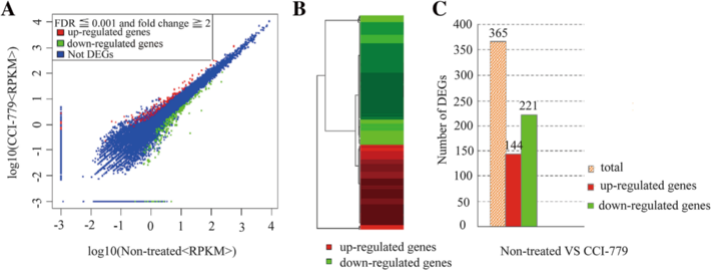
RNA sequencing data of comparison transcriptomic in untreated vs CCI-779-treated cells. **a** The spots on the MA plot represent each gene within the control and CCI-779-treated groups. The *red dots* represent upregulated genes in CCI-779-treated cells, and the *green dots* represent downregulated genes; the *blue dots* show genes not differentially expressed between groups. **b** Clustering of gene expression patterns reveals genes with functional correlations. **c** The number of DEGs in comparison transcriptomic in untreated vs CCI-779-treated cells

To analyze DEGs that were related to mTORC1, all sequenced genes were screened between control cells and treated cells (untreated vs CCI-779). A total of 365 DEGs that were related to mTORC1 were identified in the transcriptomic comparison (Fig. 2a; Additional file 2), comprising 144 upregulated and 221 downregulated DEGs (Fig. 2b, c), suggesting that their expression was associated with mTORC1 in GFb cells.

### Functional analysis of differentially expressed genes in untreated vs CCI-779-treated cells

**Fig. 3:**
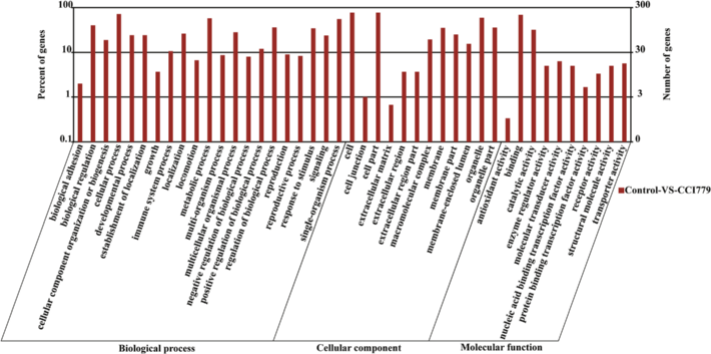
GO functional classification statistics of proteins corresponding to genes in untreated vs CCI-779-treated cells. The y-axis represents number and percent of genes, and the x-axis displays three categories by Gene Ontology: biological process, cellular component, and molecular function

To determine the functions of our DEGs, WEGO was used to attach functional annotations to them. The complete GO enrichment analysis of DEGs is included in Additional file 3. GO functional classification of DEGs was generalized as biological process, cellular process, and molecular function (Fig. 3, Additional file 4), and 300 genes were annotated with 43 GO terms. Overall, “cell (GO: 0005623) (231 genes),” “cell part (GO: 0044464) (231 genes),” and “organelle (GO: 0043226) (177 genes)” were listed as the top Cellular Components; “binding (GO: 0005488) (206 genes)” and “catalytic activity (GO: 0003824) (95 genes)” were the top Molecular Functions; and the most frequent Biological Processes were “cellular process (GO: 0009987) (215 genes),” “signal-organism process (GO: 0044699) (166 genes),” and “metabolic process (GO: 0008152) (173 genes).” Further, DEGs were enriched in KEGG terms, and 293 genes were annotated in 194 pathways, primarily “Metabolic pathways,” “Focal adhesion,” and “Regulation of actin cytoskeleton” (Additional file 5).

### CCI-779 induces expression changes in genes encoding proteins involved in mTOR signaling

**Fig. 4:**
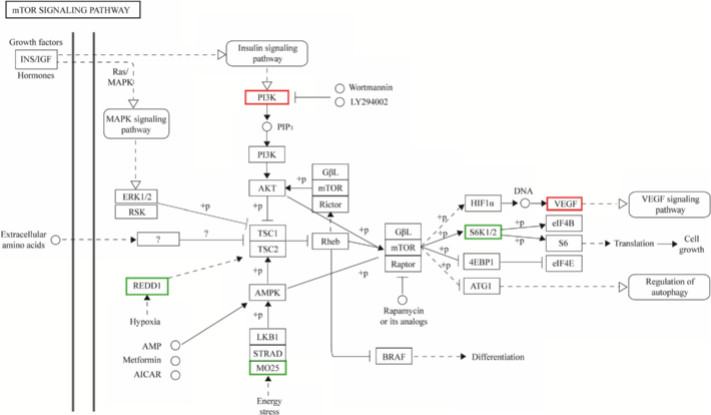
CCI-779 treatment induces expression change in mTOR signaling pathway components. Heat map data transposed onto the KEGG mTOR signaling pathway. REDD1, MO25, and S6K1/2 were downregulated (*green box*) and PI3K and VEGF (LOC102177848) were upregulated (*red box*) in CCI-779-treated GFbs (FDR ≦0.001 and fold-change ≧2)

To examine whether the proteins involved in mTORC1 signaling were differentially expressed in treated cells, the encoding genes were identified by KEGG analysis. Five genes were identified as being significantly altered in untreated versus CCI-779-treated cells—phosphatidylinositol-3-kinase (PI3K) and vascular endothelial growth factor VEGF (LOC102177848) were upregulated, and ribosomal protein S6 kinase (S6K1), regulated in DNA damage and development 1 (REDD1), and mouse protein 25 (MO25) were downregulated (Fig. 4), suggesting that inhibition of mTORC1 changes the expression of molecules that participate in this pathway.

### CCI-779 induces expression changes in eukaryotic RNA polymerase subunits and transcription factors in GFb cells

**Fig. 5:**
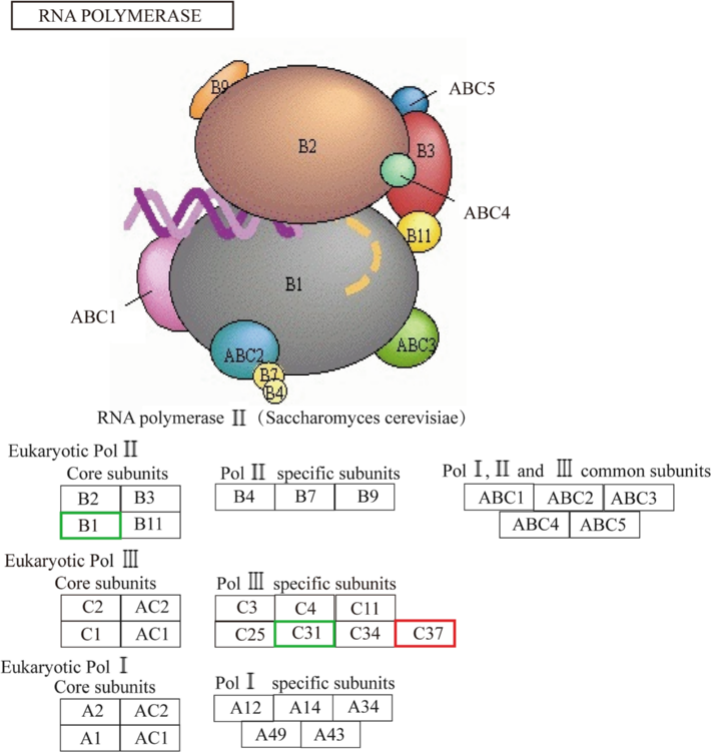
CCI-779 treatment induces expression changes in RNA polymerases subunit genes. Heat map data transposed onto the KEGG mTOR signaling pathway. The eukaryotic Pol II core subunit RPB1 (ODF3L2, B1) and Pol III specific subunit POLR3G (C31) were downregulated (*green box*), and eukaryotic Pol III specific subunit CMYA5 (C37) was upregulated (*red box*) in CCI-779-treated GFbs (FDR ≦0.001 and fold-change ≧2). The *black box* indicates no significant change in expression

To determine whether the expression of DNA-directed RNA polymerases or transcription factors were affected by mTORC1 inhibition, the expression of several eukaryotic RNA polymerase subunits and transcription factors were analyzed in control and CCI-779-treated GFbs. Eukaryotic RNA polymerase II subunit RPB1 (ODF3L2, B1) and polymerase III subunit POLR3G (C31) were downregulated, whereas CMYA5 (C37) was upregulated in CCI-779-treated GFb cells (Fig. 5). Three transcription factors were differentially expressed—TCF20 was upregulated, and NRL and NFYB were downregulated, indicating that their expression is regulated by mTOR signaling.

### Analysis of DEGs in amino acid metabolism related to mTORC1

**Fig. 6:**
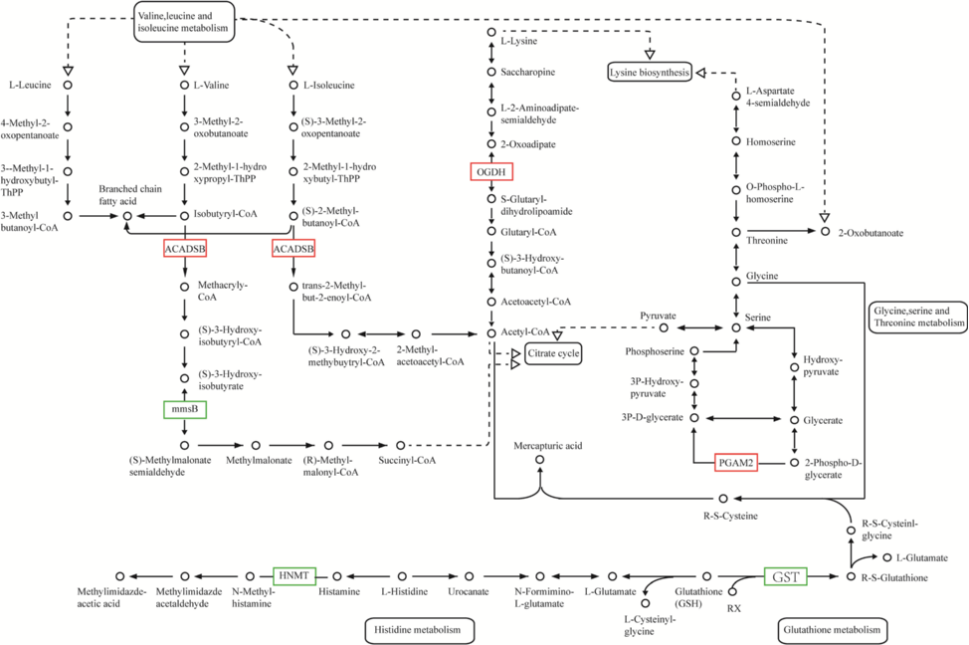
CCI-779 treatment induces expression change in amino acid metabolism enzyme genes. GST (LOC102179473, LOC102189581, LOC102188913), mmsB (PSIP1), and HNMT were downregulated (*green box*) and OGDH, ACADSB, and PGAM2 were upregulated (*red box*) in CCI-779-treated GFbs (FDR ≦0.001 and fold-change ≧2). The *black box* indicates no significant change in expression

To determine the function of mTORC1 in amino acid metabolism, a comparative analysis was performed in untreated and CCI-779-treated cells. A total of 6 genes were identified. Compared with control cells, in CCI-779-treated GFbs, 2-oxoglutarate dehydrogenase E1 component (OGDH), short/branched chain acyl-CoA dehydrogenase (ACADSB), and 2,3-bisphosphoglycerate-dependent phosphoglycerate mutase (PGAM2) were upregulated in lysine, valine, leucine, isoleucine, glycine, serine, and threonine metabolism. Glutathione S-transferase GST (LOC102179473, LOC102189581, LOC102188913), 3-hydroxyisobutyrate dehydrogenase mmsB (PSIP1), and histamine N-methyltransferase (HNMT) were downregulated in glutathione, valine, leucine, isoleucine, and histidine metabolism (Fig. 6). These data indicate that mTORC1 has a significant function in amino acid metabolism.

### Analysis of DEGs in lipid metabolism related to mTORC1

**Fig. 7:**
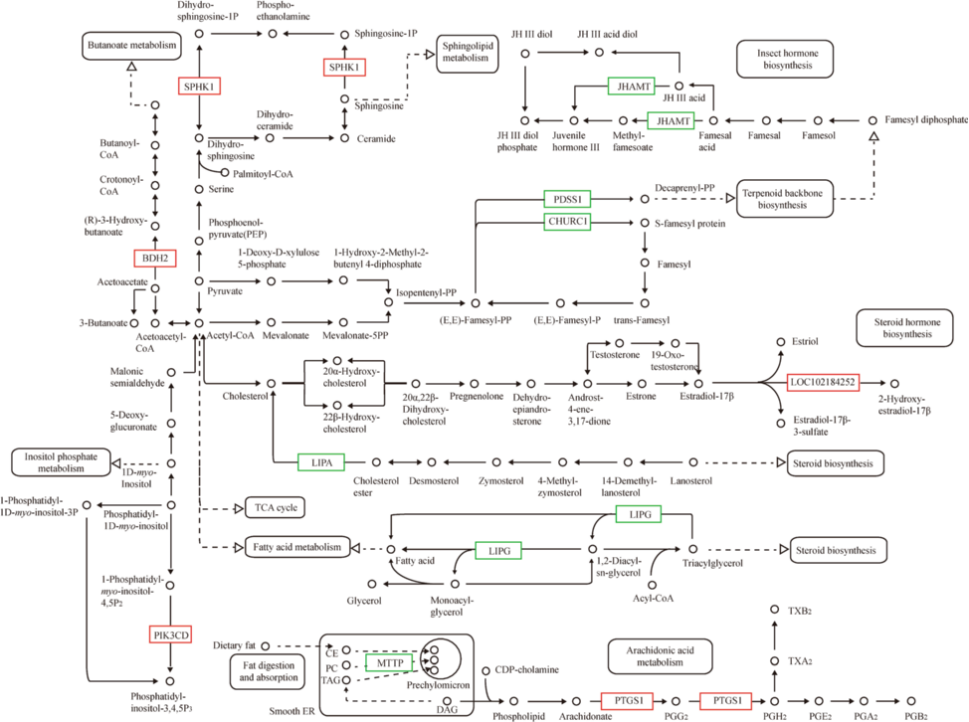
CCI-779 treatment induces expression change in lipid metabolism enzyme genes. bdh (BDH2), SPHK1, CYP 1B1 (LOC102184252), PIK3C (PIK3CD), and PTGS1 were downregulated (*green box*) and OGDH, ACADSB, and PGAM2 were upregulated (*red box*) in CCI-779-treated GFbs (FDR ≦0.001 and fold-change ≧2). The *black box* indicates no significant change in expression

To examine the regulatory mechanism of mTORC1 in lipid metabolism and identify important molecules that were related to mTORC1, we compared the expression levels of genes that were associated with lipid metabolism between CCI-779-treated and control cells. A total of 11 genes were differentially expressed in specific metabolic pathways. 3-butanediol dehydrogenase bdh (BDH2), sphingosine kinase SPHK (SPHK1), cytochrome P-450 (CYP) 1B1 (LOC102184252), phosphatidylinositol-4,5-diphosphate-3-kinase PIK3C (PIK3CD), and prostaglandin-endoperoxide synthase1 (PTGS1) were upregulated, while whereas lysosomal acid lipase LIPA (LIPA), microsomal triglyceride transfer protein large subunit (MTTP), triacylglycerol lipase ATGL (LIPG), juvenile hormone acid methyltransferase (JHAMT), protein farnesyltransferase subunit beta FNTB (CHURC1), and decaprenyl-diphosphate synthase subunit1 (PDSS1) were downregulated in treated cells (Fig. 7). BDH2, SPHK1, CYP 1B1, PIK3CD, and PTGS1 mediate butanoate metabolism, sphingolipid metabolism, steroid hormone biosynthesis, inositol phosphate metabolism and arachidonic acid metabolism, respectively. LIPA, MTTP, LIPG, JHAMT, CHURC1, and PDSS1 are involved in steroid biosynthesis, fat digestion and absorption, glycerolipid metabolism, insect hormone biosynthesis, and terpenoid backbone biosynthesis, respectively. These data suggest that mTOR signaling regulates various aspects of lipid metabolism.

### Analysis of DEGs in carbohydrate metabolism related to mTORC1

**Fig. 8:**
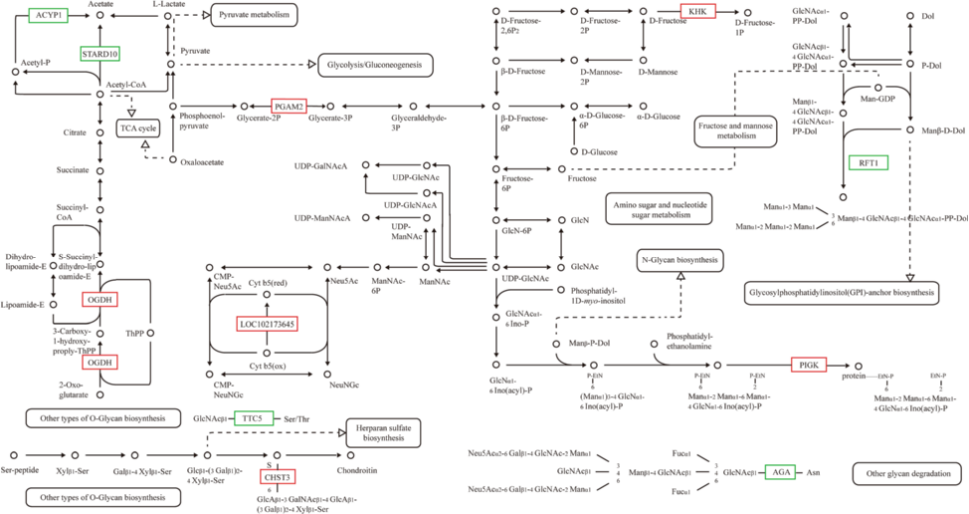
CCI-779 treatment induces expression change in carbohydrate metabolism enzyme genes. AGA, RFT1, OGT (TTC5), ACYP1, and ACH1 (STARD10) were downregulated (*green box*) while CHST3, OGDH, PIGK, PGAM2, KHK, and Cyt b5R (LOC102173645) were upregulated (*red box*) in CCI-779-treated GFbs (FDR ≦0.001 and fold-change ≧2). The *black box* indicates no significant change in expression

To analyze DEGs in energy homeostasis that were related to mTOR signaling, we focused on DEGs that mediated carbohydrate metabolism in untreated versus CCI-779-treated cells. Six genes were upregulated: chondroitin 6- sulfotransferase3 (CHST3), 2-oxoglutarate dehydrogenase E1 component (OGDH), phosphatidylinositol glycan (PIGK), 2,3-bisphosphoglycerate-dependent phosphoglycerate mutase (PGAM2), ketohexokinase (KHK), and cytochrome-b5 reductase Cyt b5R (LOC102173645). The 5 genes that were downregulated in CCI-779-treated GFbs were N4- (β- N-acetylglucosaminyl)-L-asparaginase (AGA), oligosaccharide translocation protein (RFT1), protein O-GlcNAc transferase OGT (TTC5), acylphosphatase (ACYP1), and acetyl-CoA hydrolase ACH1 (STARD10) (Fig. 8). CHST3, OGDH, PIGK, PGAM2, KHK, and Cyt b5R are involved in glycosaminoglycan biosynthesis, chondroitin sulfate biosynthesis, citrate cycle, glycosylphosphatidylinositol (GPI)-anchor biosynthesis, fructose and mannose metabolism, gluconeogenesis metabolism, and amino sugar and nucleotide sugar metabolism, respectively. AGA, RFT1, TTC5, ACYP1 and STARD10 participate in glycan degradation, N-glycan biosynthesis, other types of O-glycan biosynthesis, and pyruvate metabolism, respectively. These results indicate that carbohydrate metabolism is controlled by mTOR signaling in GFbs.

### Analysis of DEGs in single nucleotide metabolism related to mTORC1

**Fig. 9:**
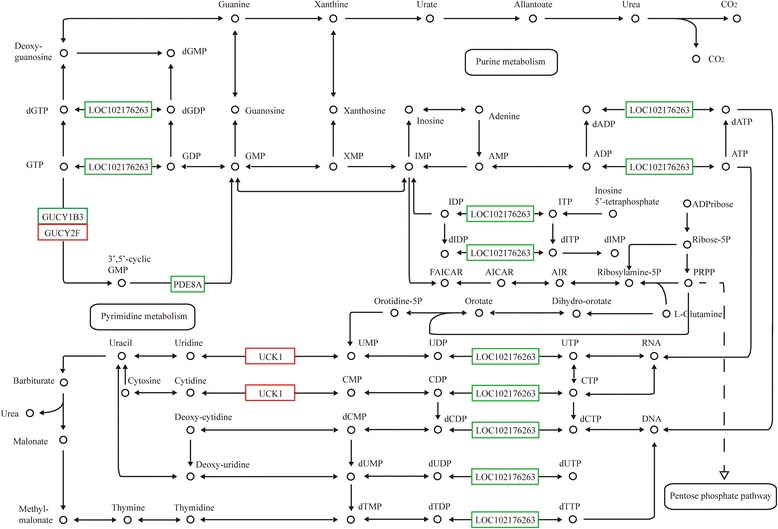
CCI-779 treatment induces expression change in single nucleotide metabolism enzyme genes. ndk (LOC102176263), GUCY1B3 and PDE8A were downregulated (*green box*) and UCK1 and GUCY2F were upregulated (*red box*) in CCI-779-treated GFbs (FDR ≦0.001 and fold-change ≧2). The *black box* indicates no significant change in expression

To determine the function of mTORC1 in single nucleotide metabolism and identify important molecules that are related to mTORC1, a comparative analysis was performed in untreated versus CCI-779 cells. In CCI-779-treated GFbs, uridine kinase udk (UCK1) and guanylate cyclase 2 F (GUCY2F) were upregulated in uridine/ cytidine and guanosine triphosphate (GTP) metabolism, respectively, whereas, guanylate cyclase soluble subunit beta (GUCY1B3), 3′,5′-cyclic-nucleotide phosphodiesterase PDE8A and nucleoside-diphosphate kinase ndk (LOC102176263) were downregulated in guanylate cyclization, 3′,5′-cyclic guanosine monophosphate (3′,5′-cGMP) and deoxy-ribonucleoside triphosphate (dNTP) metabolism, respectively (Fig. 9). These results indicate that mTOR signaling is associated with single nucleotide metabolism.

### Confirmation of gene expression

**Fig. 10:**
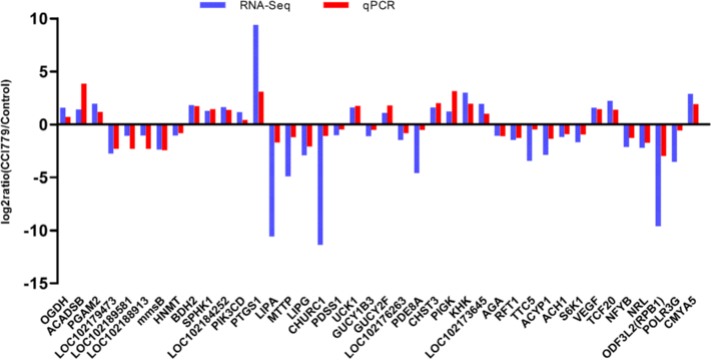
Forty differentially expressed genes (DEGs) in RNA-seq were validated by qPCR and show a high concordance. Comparison between RNA-Seq and qPCR results. X-axis shows genes validated in this study, among which some genes have different gene name and symbol, including GST (LOC102179473, LOC102189581, LOC102188913), mmsB (PSIP1), bdh (BDH2), CYP 1B1 (LOC102184252), PIK3C (PIK3CD), ATGL (LIPG), FNTB (CHURC1), udk (UCK1), ndk (LOC102176263), Cyt b5R (LOC102173645), OGT (TTC5), ACH1 (STARD10), VEGF (LOC102177848), RPB1 (ODF3L2); Y-axis shows log2ratio of expression of CCI-779 treatment versus control group. Positive value means upregulation, and negative value means downregulation. The *red column* represents qPCR results, and the *blue column* represents the RNA-Seq results. The results of the qPCR analysis for 40 genes with a significant difference and important function in amino acid, lipid, carbohydrate, and single nucleotide metabolism, RNA polymerases, transcription factors and mTOR signaling show high concordance with RNA-Seq findings

**Fig. 11:**
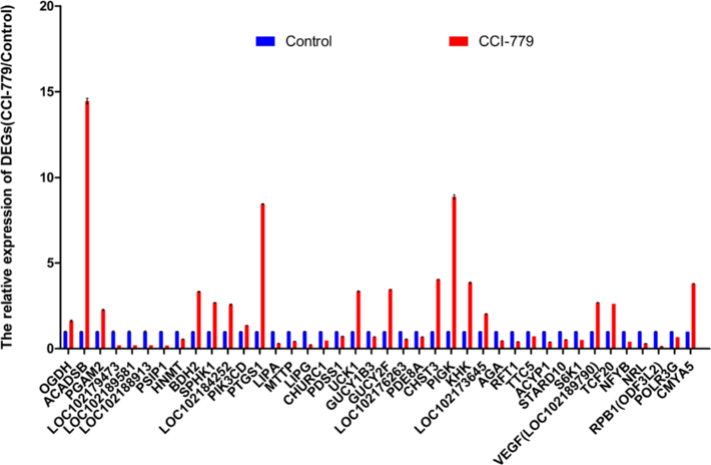
The differential expression of 40 DEGs in qPCR validation and the statistical analysis of the DEGs between control and CCI-779-treated groups. X-axis shows genes validated in this study, among which some genes have different gene name and symbol, including GST (LOC102179473, LOC102189581, LOC102188913), mmsB (PSIP1), bdh (BDH2), CYP 1B1 (LOC102184252), PIK3C (PIK3CD), ATGL (LIPG), FNTB (CHURC1), udk (UCK1), ndk (LOC102176263), Cyt b5R (LOC102173645), OGT (TTC5), ACH1 (STARD10), VEGF (LOC102177848), RPB1 (ODF3L2); Y-axis shows log2ratio of expression of CCI-779 treatment versus control group. The *blue column* represents the expression in control groups, and which value was defined as reference value (1.0). The *red column* represents the differential expression fold change of CCI-779 treatment versus control group. The qPCR data are expressed as the mean of triplicates, and error bars represent the standard deviation. Differences in means between control and CCI-779-treated groups determined by Student’s T test. The differential expression between each DEG in control and CCI-779-treated groups are all significantly different (*p* < 0.05)

**Fig. 12:**
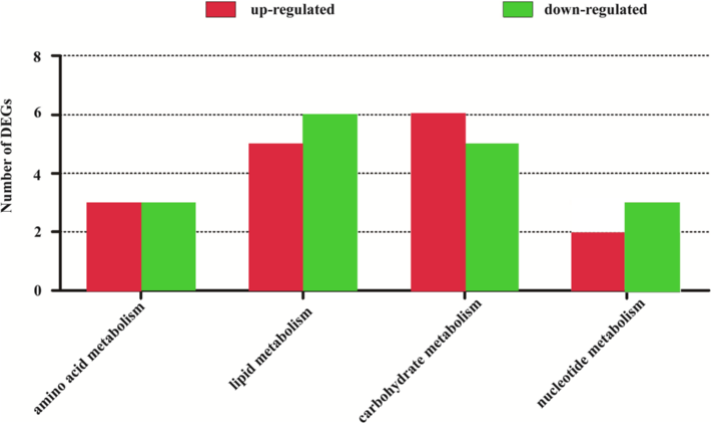
Statistics of DEGs in different macromolecule metabolism. Genes that encoded enzymes in amino acid, lipid, carbohydrate, and single nucleotide metabolism were differentially expressed in mTORC1-inhibited GFbs. Number of DEGs in lipid and carbohydrate metabolisms are more than in amino acid and nucleotide metabolism

To analyze the expression patterns of DEGs in the RNA-Seq, 40 DEGs with important functions, including 32 DEGs in amino acid, lipid, carbohydrate, and single nucleotide metabolism, 2 DEGs (VEGF and S6K1/2) involved in mTOR signaling, 3 DEGs (RPB1, POLR3G and CMYA5) related to RNA polymerase and 3 DEGs (TCF20, NRL and NFYB) related to transcription factors, were selected for quantitative real-time PCR analysis, with *β-actin* as the reference gene. The qPCR results showed high concordance with the RNA-Seq data, suggesting that our RNA-Seq findings are reliable (Fig. 10). The data are listed in Additional file 6. In addition, statistical analysis was performed for 40 DEGs, indicating that all quantitative data we achieved are reliable and the differential expression of each DEG between control and CCI-779-treated groups are all significantly different (*p* < 0.05) (Fig. 11). Moreover, genes that encoded enzymes in amino acid, lipid, carbohydrate, and single nucleotide metabolism were differentially expressed in mTORC1-inhibited GFbs. The number of DEGs in lipid and carbohydrate metabolism is more than that in amino acid and nucleotide metabolism. In addition, the number of up-regulated DEGs equals to down-regulated genes in amino acids metabolism; the number of down-regulated DEGs are more than up-regulated genes in lipids and single nucleotides metabolism; in contrast, the number of down-regulated DEGs are less than up-regulated DEGs in carbohydrates metabolism (Fig. 12). Thus, mTORC1 signaling might have disparate functions in the metabolism of various macro-molecules.

**Fig. 13:**
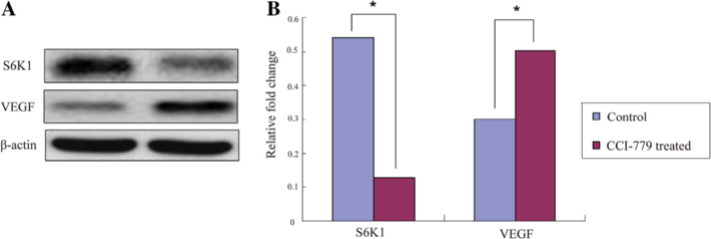
The expression of S6K1 and VEGF proteins in control and CCI-779-treated groups. S6K1 and VEGF proteins were detected by western blot analysis, which the two genes expressed differently in RNA-seq. These two proteins showed a differential expression between control and CCI-779-treated groups and a consistence with the results of the transcriptional levels. The data showed a preliminary significance of the findings of RNA-seq in the protein levels. **a** S6K1 and VEGF proteins were detected by western blot. **b** The resolved bands were quantified using Gel-Pro Analyzer 4.0 (Media Cybernetics, Inc., Rockville, MD, USA). β-actin was used as a loading control. The western blot detections of S6K1 and VEGF were carried out 3 times (Additional file 7: Figure S1) and the expression showed a significant differences (*p* < 0.05)

Emerging evidence indicates that gene transcription and steady-state levels of mRNA expression do not always predict protein levels [43]. To validate the protein expression of certain differentially expressed genes as we found in this study, S6K1 and VEGF were selected for the detection by western blot analysis. These two proteins showed a differential expression between non-treated and treated with CCI-779 groups (*p* < 0.05). The expression of S6K1 and VEGF were consistent with the results of the transcriptional levels (Fig. 13, Additional file 7: Figure S1). The data showed a preliminary significance of the findings in the protein levels.

## Discussion

The physiological and pathophysiological role of the mTOR pathway have been well-characterized in various organisms, from yeast to human [2, 3] but not in Cashmere goat. In this study, the underlying biological effect of inhibition of mTORC1 by specific chemical inhibitor CCI-779 on Inner Mongolia Cashmere goat fibroblasts was investigated, and the systematical profiling of mTOR signaling on GFbs was analyzed. mTORC1 is a central regulator of cell growth and metabolism, and is sufficient to induce specific metabolic processes [1]. mTOR signaling is emerging as a significant regulator of gene transcription, governing various cellular processes [3]. In order to investigate the regulation of mTOR signaling on key enzymes related to various metabolisms in GFbs, we present RNA-seq analysis results on cells treated with CCI-779 and untreated cells; 365 DEGs were screened out in a transcriptomic comparison of untreated versus CCI-779-treated cells. Our analysis of DEGs that are associated with mTOR signaling in eukaryotic RNA polymerase subunits, transcription factors, and macromolecule metabolism provides novel findings in GFb cells and thus constitute a valuable genetic resource on the mechanisms of mTOR signaling in regulating gene expression in the metabolism of cellular amino acids, lipids, carbohydrates, and single nucleotides.

Inner Mongolia Cashmere goat is an economically important domestic animal with the best cashmere—cashmere goats have long been an excellent local variety of cashmere and meat through natural evolution and artificial breeding. In this study, RNA-seq analysis was performed to determine the effects of CCI-779 on GFbs. CCI-779 is a specific inhibitor of mTORC1, and cells treated with CCI-779 clearly experienced significant alterations in mTORC1 signaling. In total, 144 genes were upregulated and 221 genes were downregulated, in association with mTORC1 inhibition. By cluster analysis, these DEGs were generalized as biological process, cellular component, and molecular function, including activation of RNA polymerases and transcription factors; mTOR signaling; and amino acid, lipid, carbohydrate, and single nucleotide metabolism. Our bioinformatics results were validated by qPCR analysis of 40 genes that were representative of the most relevant metabolic processes. We may infer from the RNA-seq findings that the mTOR pathway promotes accumulation of excellent cashmere and meat by regulating nutrients metabolism in cashmere goat. These findings may be helpful to improve cashmere goat breeding and its economical value or related industry.

mTORC1 has been argued to regulate the expression of genes that are transcribed by RNA polymerase II and that of transfer RNA (tRNA) and 5S rRNA through modulation of RNA polymerase (Pol) III activity [27]. mTOR kinase is required for the control of RNA polymerase III-dependent transcription and might be linked to the activities of all 3 RNA polymerases [30]. This study provides evidence that the inhibition of mTOR signaling affects the expression of eukaryotic RNA polymerase II subunit B1 and polymerase III subunits C31 and C37, indicating that mTOR signaling is involved in the transcription of RNA polymerase II-related genes, tRNA, snRNA and 5S rRNA.

In the past several years, many studies have claimed that mTOR inhibition affects transcription by regulating the activation of specific transcription factors [27, 44]. Our transcriptomic analysis revealed 3 differentially expressed transcription factors—TCF20, NRL, and NFYB—the expression of which was related to mTORC1. TCF20 (also called SPBP) is believed to be a transcriptional coactivator of NRF2, which is a master regulator of the response to oxidative stress and regulates the basal and inducible expression of many antioxidant pathway genes [45]. The depletion of TCF20 decreases the expression of the autophagy maker protein LC3B [46] and autophagy machinery [47].

The neural retina leucine zipper transcription factor NRL is essential for rod photoreceptor development [48]. The activation of rod genes in cone-like precursors depends on the induction of NRL [49]. E2F1 regulates cell proliferation and apoptosis by controlling various target genes and combines with NFYB to control the NFYB/E2F1 joint transcriptional program [50, 51]. These transcription factors are primarily associated with cell autophagy, proliferation, and apoptosis, which are regulated by mTORC1 signaling. Our data indicate that mTORC1 regulates several cellular processes.

mTOR signaling is emerging as a significant regulator of various metabolic processes through complex regulatory mechanisms [3]. It is believed that amino acids are essential for mTORC1 activity [24], but the function of mTOR in amino acid catabolism has not been reported. Our data show that the inhibition of mTOR induces significant changes in key enzymes in the metabolism of several amino acids, indicating that mTORC1 governs amino acid catabolism directly or indirectly.

Recent studies have shown that mTORC1 has a significant function in regulating various aspects of lipid metabolism in mammalian cells, including lipogenesis, adipogenesis, lipolysis, and lipid oxidation [9]. In our work, DEGs that encode important enzymes that are related to lipid metabolism were screened in CCI-779-treated GFbs, indicating that mTOR signaling has a critical function in lipogenesis and lipolysis in goat fetal fibroblasts.

mTOR has a significant function in energy homeostasis [16], including lipid and glucose metabolism. Tight and well-adapted balance of mTOR activity is required for controlling energy homeostasis under physiological conditions [52]. In addition, glucose consumption metabolically restricts T cells, leading to dampened mTOR activity and glycolytic capacity [19]. Our study has verified the presence of a series of DEGs in carbohydrate metabolism in mTORC1-inhibited cells, demonstrating an important function of mTOR signaling in energy homeostasis.

Moreover, we found that MO25, an upstream regulator of AMP-activated protein kinase (AMPK), was downregulated in CCI-779-treated cells. AMPK is a master sensor and central regulator of cellular energy status. The adaptor molecule MO25 recruits and activates LKB1 through the pseudokinase, STRAD [53]. Energy stress induces a substantial increase in AMPK activity through the LKB1/MO25/STARD complex [54]. Activated AMPK inhibits the stimulation of mTOR signaling to regulate cell metabolism and energy homeostasis, and the suppression and degradation of AMPK effect the activation of mTOR signaling [55]. Notably, the inhibition of mTOR signaling decreased the expression of MO25 in our study, suggesting that a new mechanism of mTORC1 regulation of energy homeostasis has an important function in GFb cells.

Emerging evidence suggests that mTORC1 stimulates the synthesis of single nucleotides, and mTORC1 positively regulates such anabolic processes as nucleotide biosynthesis with sufficient nutrients [5]. Through the SREBP transcription factors, mTORC1 upregulates pentose phosphate pathway (PPP) genes and thus stimulates the production of 5′-phosphoribosyl-1’-pyrophosphate (PRPP), which acts as a ribose moiety for the synthesis of purine and pyrimidine nucleotides [6]. Further, two recent studies demonstrated that mTORC1 stimulates de novo pyrimidine biosynthesis through S6K1-mediated phosphorylation and activation of CAD [7, 8]. Our study identified several DEGs that are involved in single nucleotide metabolism in CCI-779-treated cells. Pyrimidines are high-energy molecules that drive specific cellular reactions. UTP and CTP activate carbohydrates for transfer to other molecules, and CTP is an energy source for lipid synthesis. Our data indicate that mTORC1 has a significant function in this metabolic process by controlling the expression of key enzymes.

In this study, 40 significant DEGs with important function related to mTOR signaling, RNA polymerase, transcription factors and metabolism were selected for qPCR analysis or western blot analysis, and the quantitative results between the analysis methods were concordant (Figs. 10 and 13). In the qPCR analysis, the expression levels of the 40 DEGs showed a significant difference in the fold changes (*p* < 0.05) (Fig. 11). Moreover, genes that encoded enzymes in amino acid, lipid, carbohydrate, and single nucleotide metabolism were differentially expressed in mTORC1-inhibited GFbs. The number of DEGs in lipid and carbohydrate metabolism is more than that in amino acid and nucleotide metabolism. Thus, mTORC1 signaling might have disparate functions in the metabolism of various macromolecules. However, the new DEGs found in comparative transcriptome profiling on Inner Mongolia Cashmere goat fetal fibroblasts (GFbs) need to be verified and further investigated.

## Conclusions

We performed RNA-seq analysis to determine the effects of mTORC1 inhibition on the fetal fibroblast transcriptome of Cashmere goat, an important breeding line for the cashmere industry. mTORC1 inhibition induced gene expression changes that were associated with certain physiological processes. Using a digital gene expression system, 365 DEGs were identified on mTORC1 inhibition, comprising 144 upregulated genes and 221 downregulated genes compared with the control group. The DEGs were related to RNA polymerase subunits, transcription factors, mTOR kinases, and amino acid, lipid, carbohydrate, and single nucleotide metabolism, indicating that they have significant function in GFbs. The transcriptional expression of 40 DEGs that were representative for the most relevant metabolic processes, RNA polymerase, transcription factors and mTOR signaling were analyzed by qPCR and western blot, and all showed a high concordance with the RNA-Seq data. This report is the first study to indicate that mTOR signaling is an important regulator of cell metabolism, and has physiological significance in goat fetal fibroblasts and provides valuable information on metabolism in these cells.

## Additional files

Additional file 1: Table S1. Primers used in validation of DEGs. (XLSX 12 kb)
Additional file 2: Table S2. All DEGs in comparative transcriptome. (XLS 177 kb)
Additional file 3: Table S3. Gene Ontology enrichment analysis of DEGs. (XLS 611 kb)
Additional file 4: Table S4. Gene Ontology functional classification of DEGs. (XLS 98 kb)
Additional file 5: Table S5. KEGG pathway enrichment analysis of DEGs. (XLS 38 kb)
Additional file 6: Table S6. Validation data of 32 DEGs. (XLS 69 kb)
Additional file 7: Figure S1. The western blot detection of S6K1 and VEGF expression in 3 samples. Sample 1 shows the exposure of S6K1 and VEGF with the reference band of Marker (EasySee Western Marker, DM201, 25–90 kDa, TransGen Biotech, Beijing, China); Sample 2 and Sample 3 just show target bands. The bands were quantified using Gel-Pro Analyzer 4.0 (Media Cybernetics, Inc., Rockville, MD, USA). The expression of target bands in 3 samples were determined respectively, and mean ± SD was obtained. Student’s T-test indicates that the expression of S6K1 and VEGF between Control group and CCI-779 treated group all have significant differences (*p* < 0.05). (PDF 127 kb)

## Abbreviations

DEGs: Differentially expressed genes
GFbs: Goat fetal fibroblasts
GO: Gene Ontology
KEGG: Kyoto Encyclopedia of Genes and Genomes
mTOR: Mammalian target of rapamycin
qRT-PCR: Quantitative real-time polymerase chain reaction
RPKM: Reads per kb per million reads
