# Supplementary material for: A quantitative transcriptomic analysis of the physiological significance of mTOR signaling in goat fetal fibroblasts
Source: BMC Genomics. 2016 Nov 7;17:879. doi: 10.1186/s12864-016-3151-y (PMC5098276; doi:10.1186/s12864-016-3151-y)
Supplement: Additional file 7: Figure S1. — The western blot detection of S6K1 and VEGF expression in 3 samples. Sample 1 shows the exposure of S6K1 and VEGF with the reference band of Marker (EasySee Western Marker, DM201, 25–90 kDa, TransGen Biotech, Beijing, China); Sample 2 and Sample 3 just show target bands. The bands were quantified using Gel-Pro Analyzer 4.0 (Media Cybernetics, Inc., Rockville, MD, USA). The expression of target bands in 3 samples were determined respectively, and mean ± SD was obtained. Student’s T-test indicates that the expression of S6K1 and VEGF between Control group and CCI-779 treated group all have significant differences (p < 0.05). (PDF 127 kb) [file 12864_2016_3151_MOESM7_ESM.pdf]

Supplementary figures

Sample 1

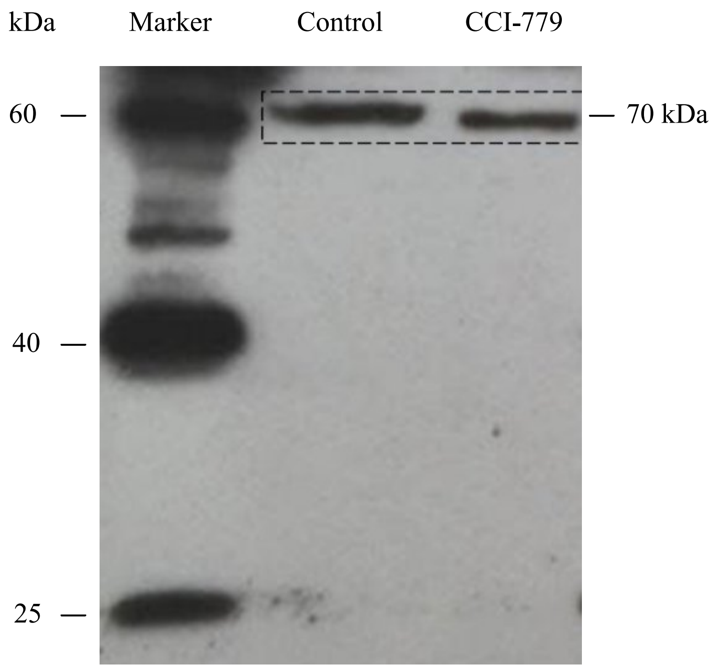

S6K1

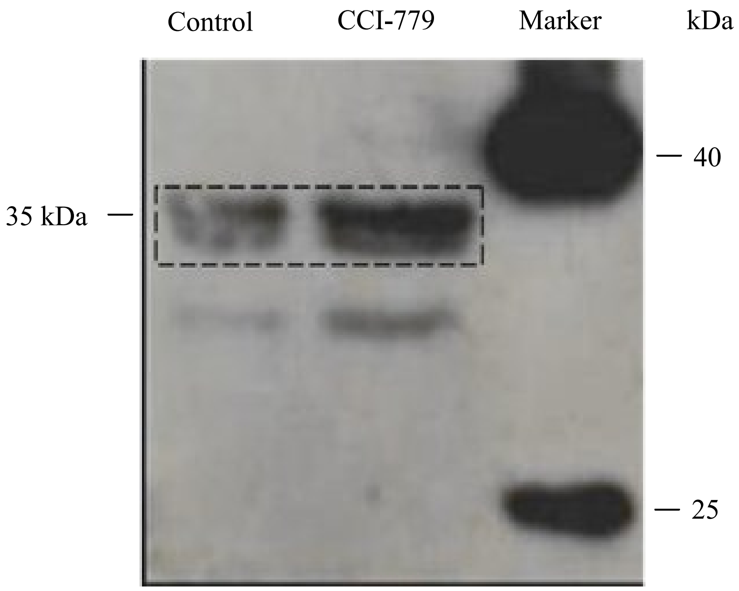

VEGF

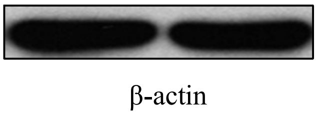

Sample 2

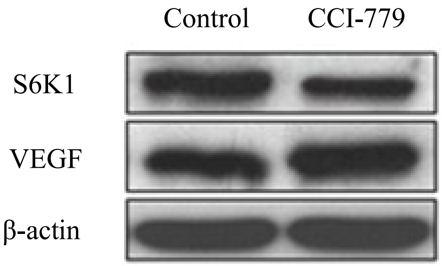

Sample 3

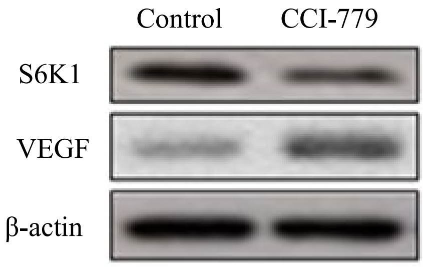

Figure.S1 The western blot detection of S6K1 and VEGF expression in 3 samples. Sample 1 shows the exposure of S6K1 and VEGF with the reference band of Marker (EasySee Western Marker, DM201, 25-90 kDa, TransGen Biotech, Beijing, China); Sample 2 and Sample 3 just show target bands. The bands were quantified using Gel-Pro Analyzer 4.0 (Media Cybernetics, Inc., Rockville, MD, USA). The expression of target bands in 3 samples were determined respectively, and mean±SD was obtained. Student's T-test indicates that the expression of S6K1 and VEGF between Control group and CCI-779 treated group all have significant differences ($p < 0.05$).
